# Low Doses of Melatonin to Improve Sleep in Children with ADHD: An Open-Label Trial

**DOI:** 10.3390/children10071121

**Published:** 2023-06-28

**Authors:** Ana Checa-Ros, Antonio Muñoz-Hoyos, Antonio Molina-Carballo, Iris Viejo-Boyano, Maricarmen Chacín, Valmore Bermúdez, Luis D’Marco

**Affiliations:** 1 Grupo de Investigación en Enfermedades Cardiorrenales y Metabólicas, Departamento de Medicina y Cirugía, Facultad de Ciencias de la Salud, Universidad Cardenal Herrera-CEU, CEU Universities, Calle Santiago Ramón y Cajal s/n, Alfara del Patriarca, 46115 Valencia, Spain; luis.dmarcogascon@uchceu.es; 2 Aston Institute of Health & Neurodevelopment, School of Life & Health Sciences, Aston University, The Aston Triangle, Birmingham B4 7ET, UK; 3 Departamento de Pediatría, Facultad de Medicina, Universidad de Granada, Avda. De La Investigación 11, 18016 Granada, Spain; amunozh@ugr.es (A.M.-H.); amolinac@ugr.es (A.M.-C.); 4 Departamento de Nefrología, Hospital Universitari I Politècnic La Fe, Avda. Fernando Abril Martorell 106, 46026 Valencia, Spain; ivb_1993@hotmail.com; 5 Facultad de Ciencias de la Salud. Barranquilla, Universidad Simón Bolívar, Barranquilla 080002, Colombia; maricarmen.chacing@unisimon.edu.co (M.C.); valmore.bermudez@unisimon.edu.co (V.B.)

**Keywords:** ADHD, melatonin, sleep, actigraphy, methylphenidate

## Abstract

Objective. Only a few studies assessing the sleep effects of low doses of melatonin (aMT) have been performed in the past, most of them in adults, and only one in subjects with attention-deficit/hyperactivity disorder (ADHD). The aim of this study was to provide evidence of the changes induced by aMT doses as low as 1 mg in the sleep pattern of pediatric patients with ADHD under treatment with methylphenidate (MPH). Methods. Children and adolescents (7–15 years) with ADHD who were receiving extended-release MPH were recruited. A seven-week sleep diary was collected prior to starting a four-week treatment with 1 mg of aMT (30 min before bedtime). Seven-day actigraphic assessments of sleep were performed before and after treatment. Results. Twenty-seven patients (17 males, 62.96%) participated in the study, who had been receiving MPH for 1.57 (1.11) months. A significant increase in sleep duration (TST) was observed after one month of treatment (463 (49) min to 485 (41) min; *p* < 0.040), with nonsignificant improvements in sleep-onset latency (SOL), nocturnal awakenings, or sleep efficiency. Only minor adverse effects were reported. Conclusion. Low doses of melatonin (1 mg) are able to increase TST in children and adolescents with ADHD receiving treatment with psychostimulants, with an adequate tolerability profile. Further placebo-controlled trials adjusting the time of aMT administration to the individual circadian profile should explore the effects of low doses of this hormone to shorten SOL in this population of patients.

## 1. Introduction

Attention-deficit and/or hyperactivity disorder (ADHD) is characterized by the appearance of impairing symptoms of inattention, hyperactivity, and impulsivity in children under 12 years of age [1,2]. It represents the most common neurodevelopmental disorder, accounting for around 5% of individuals younger than 18 years [3] and variably persisting into adulthood in around 40–60% of cases [4]. According to the DSM-5 criteria, distinct types of ADHD presentations are distinguished based on the predominant core symptom (inattentive, hyperactive, or combined) [2]. Disruptions in the catecholaminergic neurotransmission system, mainly involving the dopamine (DA) and norepinephrine (NE) neurotransmitters, seem to be at the neurobiochemical bases of this disorder, eventually leading to deficits in the executive functions (attention, working memory, behavioral inhibition, planning...) [5,6,7,8,9]. Current therapies for ADHD include psychotherapy and pharmacological treatments for moderate-to-severe cases [10]. Psychostimulants, and the different formulations of methylphenidate (MPH) in particular, continue to be the first-line medication for ADHD. MPH blocks presynaptic DA and NE transporters to inhibit DA and NE reuptake into the neurons, thus facilitating dopaminergic and catecholaminergic neurotransmission [11,12,13]. However, the long-term use of MPH is often limited by their low adherence and tolerability rates, derived from the combination of frequent adverse effects, ADHD-related stigma, and social resistance to medication [14,15], particularly in adolescents [16].

The severity of ADHD and its burden for patients and families are not only due to the nuclear symptoms, but to the associated comorbidities as well [17]. Among them, sleep problems acquire special relevance. They may affect up to 70% of patients with ADHD [18], undermining their cognitive, behavioral, and physical states [19], as well as increasing parental stress levels [20]. Patients with ADHD suffer from variable sleep disruptions, but the most consistent finding is a delayed circadian phase (evening preference), with a consequent disturbance in daytime functioning [21,22].

On the other hand, sleep-onset insomnia (SOI) is one of the most common side effects classically attributed to psychostimulants, particularly to the extended-release formulations of MPH [23,24,25]. Although recent clinical trials found an improvement or, at least, no deterioration in the sleep pattern after initiating MPH treatment [26,27,28,29], what seems certain is that adverse outcomes on sleep derived from psychostimulants depend on several factors, both intrinsic (the body mass index, presence of preexisting sleep conditions, frontostriatal structural connectivity) and extrinsic to the patient (dosage optimization) [30,31,32,33].

The exact neurobiological mechanisms predisposing patients with ADHD to suffer from sleep disorders remain unknown, but they seem related to a circadian dysfunction in which the dim light melatonin onset (DLMO) is significantly delayed [34,35]. Therefore, the nighttime administration of exogenous melatonin (aMT) is a common therapeutic resource to advance the DLMO and correct these sleep/wake cycle alterations with a suitable safety profile for pediatric populations [36,37]. aMT has proved effective to reduce sleep-onset latency (SOL) and increase total sleep time (TST) in healthy children and in those with neurodevelopmental disorders, either at a fixed dose or allowing dose escalation [38,39,40]. Currently, there are no specific guidelines on melatonin dosage in pediatric ages, except for a consensus document published by Bruni et al. [36] in 2015, who recommended a maximum dose of 3 mg and 5 mg per night in healthy children and adolescents, respectively. In a recent registry-based cohort study analyzing the prescription of aMT to the Nordic pediatric population between 2006 and 2017, the average dose ranged between 3 and 4 mg/day [41]. However, beneficial effects in ADHD have been reported with higher aMT doses, oscillating between 3 and 10 mg/night [36,42,43].

The prescription of aMT, most particularly to pediatric patients, has experienced nearly a tenfold increase in several European countries in an 11-year period [41,44]. This, together with the lack of specific guidelines on aMT dosage in children with neurodevelopmental disorders and the scarcity of double-blind randomized placebo-controlled trials [45], has raised concerns about the potential current misuse of aMT and the long-term adverse effects derived from this hormone [46,47]. Although the administration of aMT to children has traditionally been considered safe [36], it is worth noticing a lack of consistency between studies in the approaches taken to measure adverse events in patients with neurological disabilities [48]. In addition, pediatric hospitalizations and serious outcomes have recently been reported after accidental ingestions of aMT [49]. Therefore, arguing for the administration of the minimum effective dose of aMT in pediatric patients, particularly in those with neurodevelopmental disorders, seems cautious and advisable [50].

Only a few studies assessing the sleep effects of low doses of aMT have been performed in the past: most of them were conducted in adults, and only one in subjects with ADHD [51,52,53,54]. The aim of this study was to provide evidence of the changes induced by low doses of MT in the sleep pattern of pediatric patients with ADHD under treatment with psychostimulants.

## 2. Material and Methods

### 2.1. Design

This is a four-week duration, single-center, open-label clinical trial on children and adolescents with ADHD receiving extended-release formulations of psychostimulants. The primary objective was to evaluate changes in the actigraphy parameters of sleep (SOL, TST, wake time after sleep onset or WASO, and sleep efficiency or SE) before and after initiating treatment with aMT.

### 2.2. Population

Children and adolescents, between 7 and 15 years old, diagnosed with ADHD in accordance with the DSM-5 criteria [2] who had recently started treatment with a prolonged-release formulation of MPH (Concerta^®^, Janssen Pharmaceuticals, Inc., Titusville, NJ, USA), were invited to participate in the study. They were recruited between March 2015 and July 2016 from the Neuropediatrics Unit at San Cecilio University Hospital (Granada, Spain), where a primary diagnosis of ADHD was formally made after ruling out other processes leading to attention-deficit/hyperactivity symptoms as per psychometric scales, neuroimaging, blood and neurophysiological tests (electroencephalogram). Informed consent and assent were respectively gathered from parents/guardians and patients before inclusion.

The exclusion criteria were: (1) patients suffering from heart disease; (2) patients diagnosed with glaucoma; (3) presence of neuropsychiatric, metabolic, or endocrine disorders able to justify the current symptoms; (4) current intake of melatonin; (5) concomitant intake of any sleep-disturbing medication except for MPH; and/or ((6) refusal to participate.

### 2.3. Procedures

#### 2.3.1. Sleep Diary

Patients and parents/guardians were initially asked to complete the official sleep diary validated by the National Sleep Foundation (US) [55] for a seven-day period before initiating treatment with aMT. The sleep diary was intended to provide clinicians with a general overview of their patients’ sleep habits, so that sleep hygiene recommendations could reasonably be made as part of the sleep disorder management. The everyday information collected in the diary is shown in Table 1.

#### 2.3.2. Actigraphy

Patients were instructed to wear the MotionWatch 8^®^ actigraph (Cambridge Neurotechnology Ltd., Cambridge, UK) on the nondominant wrist 24 h per day for seven consecutive days at baseline and after one month of treatment. They were requested to only remove the device for sport activities and showers/baths (nonwaterproof), as well as to press the “event marker” button when they switched the lights off at night and got up in the morning. Recordings of the amount of activity were made at 30 sec epochs, which were analyzed with the MotionWare^®^ software version 1.1.20 (Cambridge Neurotechnology Ltd., Cambridge, UK). This software, by classifying epochs as “sleep” or “awake” using a validated algorithm, calculates the following sleep parameters: light-off time (hrs:min); sleep-onset time or SOT (the clock time when the patient fell asleep, expressed in hrs:min); wake-up time in the morning (hrs:min); SOL (the time between light-off and SOT, expressed in min); TST (total time categorized as “sleep” between SOT and wake-up time, expressed in min); WASO (total time categorized as “awake” between SOT and wake-up time, expressed in min); and SE (total time spent sleeping calculated as a percentage of the difference between light-off and get-up times).

#### 2.3.3. Therapeutic Intervention

Patients were instructed to take 1 mg of fast-release aMT (around 30 min before the usual bedtime) for one month once the baseline data from the sleep diary and actigraphy were obtained. The aMT was specifically prepared at the hospital pharmacy.

**Figure 1:**
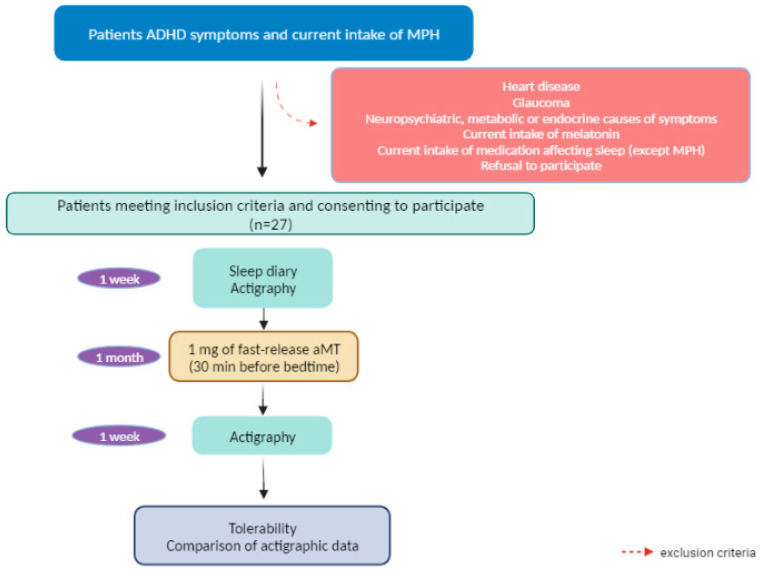
Schematic representation of the study protocol. Abbreviations: ADHD: attention-deficit/hyperactivity disorder; MPH: methylphenidate; aMT: melatonin. Created with BioRender.com.

A diagram of the study is shown in Figure 1.

### 2.4. Ethical Aspects

The study protocol was approved by the Ethics Committee of Biomedical Research at San Cecilio University Hospital (Granada, Spain). All procedures were carried out in accordance with the Declaration of Helsinki as revised in 2013 [56].

### 2.5. Statistical Analysis

Descriptive data were presented as mean and standard deviation (SD). For comparative analyses, the Kolmogorov–Smirnov test was applied to verify the normality assumption before running a Student *t* test to make comparisons between the actigraphic parameters before/after treatment. The significance level was set at α = 0.05. The Statgraphics Centurion XVII software version 17 (Statpoint Technologies, Inc., Warrengton, VA, USA) was used for statistical calculations.

Considering a 5% difference in total sleep length as clinically relevant, for an ADHD prevalence of 5%, assuming a power of 80% and an alpha of 0.05, the minimum sample size obtained was 26.5 patients.

## 3. Results

### 3.1. Population

Twenty-seven children and adolescents with ADHD participated in this study. The sample was composed of 27 patients, 17 males (62.96%) and 10 females (37.04%), aged 7 to 15 years, with a mean age of 9.67 (2.13) years. In accordance with the DSM-5 criteria [2], 14 patients (51.85%) were classified as a predominantly inattentive presentation (ADHD-I), 12 of them (44.44%) as a combined presentation (ADHD-C) and only one patient (3.70%) was considered as predominantly hyperactive/impulsive (ADHD-HI). Patients had been receiving treatment with Concerta^®^ (Janssen-Cilag S.A., Madrid, Spain) in the morning at a daily dose between 0.7 and 1 mg/kg for a mean period of 1.57 (1.11) months. Associated comorbidities had been detected in 15 participants (55.55%), predominantly oppositional defiant disorder, anxiety, and fine motor skill disorder.

The demographic and clinical characteristics of our participants are shown in Table 2.

### 3.2. Sleep Diary

According to the information collected in the sleep diary, patients fell asleep an average of 11.26 (6.09) min after going to bed. The reported mean TST was 8.04 (0.94) hours. Difficulties in falling asleep, with a reported SOL > 15 min, were reported in four patients (14.81%). The total amount of sleep was reported as <8 h in eight patients (29.63%), half of whom were adolescents. Three or more nocturnal awakenings were reported in four patients (14.81%). Three subjects (11.11%) reported waking up feeling refreshed less often than four days/per week. The majority of our patients (66.66%) reported watching television as the activity performed before going to bed. Sleep disturbance was reported by one patient (3.70%) as “sleep-talking” (somniloquy) (Table 3).

### 3.3. Actigraphy

The most remarkable result after one month of treatment with aMT was a significant increase in the mean TST, from 463 (49) min to 485 (41) min (*p* < 0.040). Nonsignificant improvements were observed for the other parameters after treatment, such as the SOL (11 (11) min vs. 10 (8) min (*p* = 0.699)), WASO (72 (44) min vs. 71 (44) min, *p* = 0.933), and the SE (86.70 (7.13)% vs. 88.66 (6.24)%, *p* = 0.271) (see Table 4).

### 3.4. Tolerability

No severe adverse events were reported. Mild and transient gastric discomfort and anorexia were reported by 12 patients (44.44%).

Overall, parents/guardians reported an improvement in sleep latency and total sleep duration after treatment with aMT, with no noticeable effect on nocturnal awakenings.

## 4. Discussion

In this open-label clinical trial conducted on children and adolescents with ADHD receiving treatment with MPH, we explored the changes in the sleep parameters as measured by actigraphy after administering a low dose of aMT (1 mg/night) during four weeks. The most noticeable result was a significant increase in the TST with a nonsignificant reduction in the SOL, accompanied by minor improvements in other sleep values, such as the SE and WASO. In all patients, aMT was well tolerated, and only minor and transient adverse events were reported.

Only a few studies have explored the effects on sleep provoked by doses of aMT lower than usual; most of them have been conducted in adults suffering from sleep-onset insomnia. In this regard, the first report was made by James et al. [51] in 1990. Through a double-blind design, participants were randomized to receive either 5 mg or 1 mg of aMT for one week. Changes in the sleep pattern were assessed through a sleep electroencephalogram (EEG). Of note, no changes were observed in either the SOL or the TST, except for an increase in the rapid-eye-movement latency with the lowest dose. In 1995, Haimov et al. [57] explored the actigraphic sleep values derived from the administration of low doses of two different formulations of aMT, fast-release (FR) and sustained-release (SR), in a population of elderly people suffering from insomnia. The study was divided into two periods: during the first period, a double-blind placebo-controlled trial was performed and patients were randomized to receive 2 mg of FR-aMT, 2 mg of SR-aMT, or a placebo for one week; in the second period, patients were administered 1 mg of SR-aMT for two months. In all cases, aMT was taken 2 h before the desired bedtime. The effects observed on the different actigraphic values were formulation-dependent: changes in the SOL were due to the FR-aMT, as this presentation provoked a sudden increase in aMT levels immediately after administration; however, the SR-aMT had a higher impact on the SE and sleep maintenance by ensuring the continuous release of small amounts of aMT throughout the entire night. In contrast to Hasimov et al. [57], reductions in the SOL were reported by Hughes et al. [58] after two weeks of treatment with 0.5 mg of both FR-aMT and SR-aMT in a sample of adults with age-related insomnia (55–80 years old). However, no changes were observed in the TST. As in our study, 30 min before the usual bedtime was the timing established by Hughes et al. [58] for aMT administration.

Further authors attempted to establish an association between the changes in the sleep parameters and the modifications in peripheral aMT levels. In a placebo-controlled trial, Dawson et al. [59] reported a significant increase in the nocturnal urinary excretion of 6-sulphatoxymelatonin (aMT-6S) after administering transbuccal aMT at 0.5 mg to adults with sleep maintenance insomnia for four consecutive nights. aMT was instructed to be taken 2 h before going to bed. However, the changes in aMT-6S did not translate into any significant finding in the polysomnographic (PSG) sleep parameters. The most relevant investigation in this regard was carried out by the group of Zhdanova et al. [52] through a double-blind placebo-controlled trial on a sample of adults over 50 years old with chronic insomnia. Changes in PSG sleep parameters and serum aMT levels were explored after subjects had been randomized to receive three different doses of aMT (0.1 mg, 0.3 mg, and 3 mg) 30 min before bedtime for a nine-week period. The dose of 0.3 mg of aMT was the one achieving the greatest improvement in sleep efficiency (considered as the amount of TST in the period between light-off time and get-up time), accompanied by a restoration of the aMT circadian profile. No significant changes in the SOL were documented by the authors. In contradiction with Zhdanova et al. [52], Almeida-Montes et al. [53] reported no significant changes in sleep measures after administering aMT at either 0.3 mg or 1 mg to a small sample (*n* = 10) of adults with primary insomnia in a later double-blind placebo-controlled study with a crossover design. Nonetheless, methodological differences could account for the divergences found between studies, such as the study period and the measures to assess sleep: sleep EEG and sleep logs were used to evaluate sleep after a seven-day treatment in the Almeida-Montes’ study.

The only clinical trial investigating the results obtained with low doses of aMT in patients with ADHD was performed by van Andel et al. [54] in 2020 on adults with ADHD (21–40 years old) and delayed sleep phase syndrome (DSPS). DSPS is a circadian rhythm disorder particularly common in adolescents and young adults, in which the delay of the sleep phase causes difficulty in falling asleep and waking up at a desired time [60], due to a circadian delay in DLMO [61]. Participants were randomized to one of the three treatment arms for three weeks: (1) placebo; (2) aMT at 0.5 mg/night; or (3) aMT (0.5 mg) plus bright light therapy (BLT). The primary outcome was the advancement of the DLMO as measured in saliva. More than 1 h of advancement in the DLMO was reported for the patients receiving aMT and aMT + BLT, with no significant differences between these two groups. No results in the TST were revealed. Unlike our study, in van Andel’s trial, the intake of MPH was an exclusion criterion, and the time of aMT administration was not set at an established fixed hour but adjusted to the individual DLMO (3 h before). Nonetheless, differences in the sleep pattern and comorbidities between adult and pediatric ADHD populations must be considered when making comparisons between van Andel’s and our study.

Regarding pediatric populations, Eckerberg et al. [62] performed a five-week duration double-blind crossover study in which 20 adolescents (14–19 years old) with DSPS were randomized to receive either a placebo or aMT (1 mg) in alternate weeks. The administration of aMT/placebo took place in the timeframe between 16:30 h and 18:00 h. A significant reduction in the SOL with a significant increase in the TST was observed with aMT at 1 mg compared with a placebo. This clinical finding was supported by a recovery of the circadian levels of aMT in saliva. The improvement in the TST with 1 mg of aMT in pediatric patients is something we also observed in our study, even though the time for aMT administration and the sleep measures used (the Karolinska sleepiness scale) in Eckerberg’s study differed from the present trial.

Given the heterogeneity between trials in relation to the dosage and time of aMT administration, population, study duration, and sleep measures, making comparisons between studies seems arduous, as reported by a prior systematic review attempting to standardize a set of outcome measures for sleep intervention studies [48]. The improvement that we obtained in the actigraphic TST with a low dose of aMT was previously corroborated by studies using objective and subjective sleep measures, such as those by Zhdanova et al. [52] and Eckerberg et al. [62]. More striking was that the SOL barely changed in our study, as this finding does not support prior evidence stating that low doses of aMT (≤1 mg) may be more effective at initiating sleep and reducing the SOL [63]. However, higher doses of aMT would show more soporific effects and act on the TST more specifically [63,64]. We attribute these discrepancies to the dosing time of aMT in our study: although the use of aMT 30 min before the desired bedtime was traditionally considered in previous investigations [52,59], current evidence suggests the administration at least 2 h before the individual DLMO when it refers to doses as low as 1 mg [62]. In the absence of DLMO, the administration of aMT between 3 and 4 h before the actual sleep-onset time, as recommended by Bruni et al. [36], would have constituted a better approximation to the actual aMT secretion profile of patients in our study.

Our study had several limitations. First, a double-blind randomized placebo-controlled design would have minimized the probability of performance bias. Second, measurements of peripheral aMT levels (such as the DLMO in saliva) would have allowed a better optimization of aMT effects by adapting the dosing time to the patient’s actual secretion profile. Third, the majority of patients reported watching television before going to bed, which could have influenced the results observed in our sample, given the diminishing effects of screen exposure on aMT production [65,66]. Fourth, comparisons in the sleep diary data before and after aMT administration were not allowed, as a second seven-day sleep diary was not completed after therapy. Finally, the wide age range of our sample (7–15 years) could account for divergent and heterogeneous sleep values in our sample influencing the effects of aMT, particularly considering the hormonal changes and the DSPS phenomenon in adolescents.

To date, this is the first study reporting on the sleep effects obtained with low doses of aMT in pediatric patients with ADHD under treatment with psychostimulants. We have managed to assess the sleep pattern of ADHD subjects within the realistic context of concomitant treatment with psychostimulants, as well as to provide the information through actigraphy as the most suitable objective sleep measure [48]. Further randomized placebo-controlled trials adjusting the time of administration to the individual DLMO are required in children and adolescents with ADHD to confirm these results.

## 5. Conclusions

Low doses of melatonin (1 mg) are able to increase the sleep length in children and adolescents with ADHD while receiving treatment with psychostimulants, with an adequate tolerability profile. Further placebo-controlled trials adjusting the time of melatonin administration to the individual circadian profile should explore the effects of low doses of this hormone to advance the DLMO in this population of patients.

## Figures and Tables

**Table 1:** Information collected in the sleep diary.

Completing at wake-up time
(1) Bedtime (2) Sleep onset (3) Night awakenings (4) Get-up time (5) Feeling still tired, somewhat awake, or wide awake at wake-up time (6) Sleep duration (7) Sleep disturbances: any mental, emotional, physical, or environmental factors affecting sleep (e.g., stress, snoring, physical discomfort, temperature)
Completing at bedtime
(8) Physical exercise (at least 30 min) (9) Alcohol or heavy meal intake 2–3 h before going to bed (10) Caffeinated drink consumption (11) Medication intake (12) Activity carried out 1 h before going to sleep (watch TV, work, read...)

Extracted from: Sleep Diary. The National Sleep Foundation (Internet). 2021 (cited 11 March 2023). Available from: https://www.thensf.org/ (accessed on 10 March 2023).

**Table 2:** Demographic and clinical information of participants.

	ADHD group (*n* = 27)
Age: mean (SD) in years	9.67 (2.13)
Sex distribution:	
- Males (%)	17 (62.96)
- Females (%)	10 (37.04)
ADHD presentation:	
- ADHD-I (%)	14 (51.85)
- ADHD-C (%)	12 (44.44)
- ADHD-HI (%)	1 (3.70%)
Period taking MPH: mean (SD) in months	1.57 (1.11)
Comorbidities:	
- ODD (%)	5 (18.52)
- Conduct disorder (%)	1 (3.70)
- Anxiety (%)	4 (14.81)
- Fine motor skill disorder (%)	3 (11.11)
- Tics (%)	1 (3.70)
- Enuresis (%)	1 (3.70)

Abbreviations: SD = standard deviation; ADHD = attention-deficit/hyperactivity disorder; % = percentage; ADHD-I = predominantly inattentive presentation of ADHD; ADHD-C = combined presentation of ADHD; ADHD-HI = predominantly hyperactive/impulsive presentation of ADHD; MPH = methylphenidate; ODD = oppositional defiant disorder.

**Table 3:** Data from the sleep diary.

SOL: mean (SD) in minutes	11.26 (6.08)
TST: mean (SD) in hours	8.04 (0.94)
Nocturnal awakenings:	
- <3/night (%)	23 (85.18)
- ≥3/night (%)	4 (14.81)
Waking up refreshed:	
- Every day (%)	8 (29.63)
- <7 and ≥4 days/week (%)	16 (59.26)
- <4 days/week (%)	3 (11.11)
Activity before going to sleep:	
- Watch TV (%)	18 (66.66)
- Play (%)	4 (14.81)
- Read (%)	3 (11.11)
- Homework (%)	2 (7.41)
Caffeinated drink consumption:	
- Never (%)	23 (85.18)
- In the morning (%)	2 (7.41)
- In the evening (%)	2 (7.41)

Abbreviations: SOL = sleep-onset latency; SD = standard deviation; TST = total sleep time; % = percentage; TV = television.

**Table 4:** Comparative analysis of actigraphic data before and after treatment with melatonin.

Actigraphic Sleep Parameters	Before Treatment Mean (SD)	After Treatment Mean (SD)	*p*-Value
Light-off time (h:min)	22:59 (01:05)	23:02 (01:01)	0.897
Sleep onset (h:min)	23:11 (01:10)	23:08 (01:04)	0.891
Wake-up time (h:min)	07:56 (00:48)	08:06 (00:54)	0.561
Get-up time (h:min)	08:05 (00:42)	08:08 (00:54)	0.886
SOL (min)	11 (11)	10 (8)	0.699
TST (min)	463 (49)	485 (41)	0.040 *
WASO (min)	72 (44)	71 (44)	0.933
SE (%)	86.70 (7.13)	88.66 (6.24)	0.271

* *p* < 0.05; Abbreviations: SD = standard deviation; h = hours; min = minutes; SOL = sleep-onset latency; TST = total sleep time; WASO = awakenings after sleep onset; SE = sleep efficiency.

## Data Availability

Anonymized data related to the current study are available from the corresponding author, upon reasonable request.
